# Supplementary figures and images for: Persistent PKA activation redistributes NaV1.5 to the cell surface of adult rat ventricular myocytes
Source: J Gen Physiol. 2024 Jan 16;156(2):e202313436. doi: 10.1085/jgp.202313436 (PMC10791559; doi:10.1085/jgp.202313436)

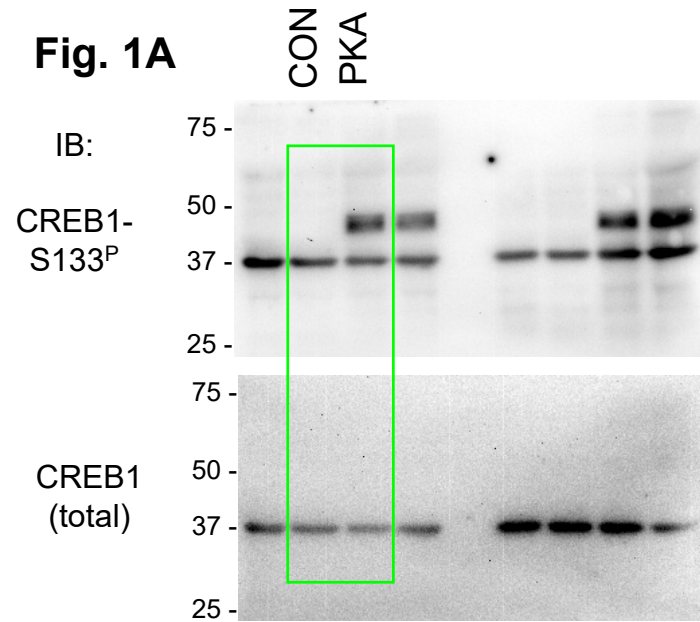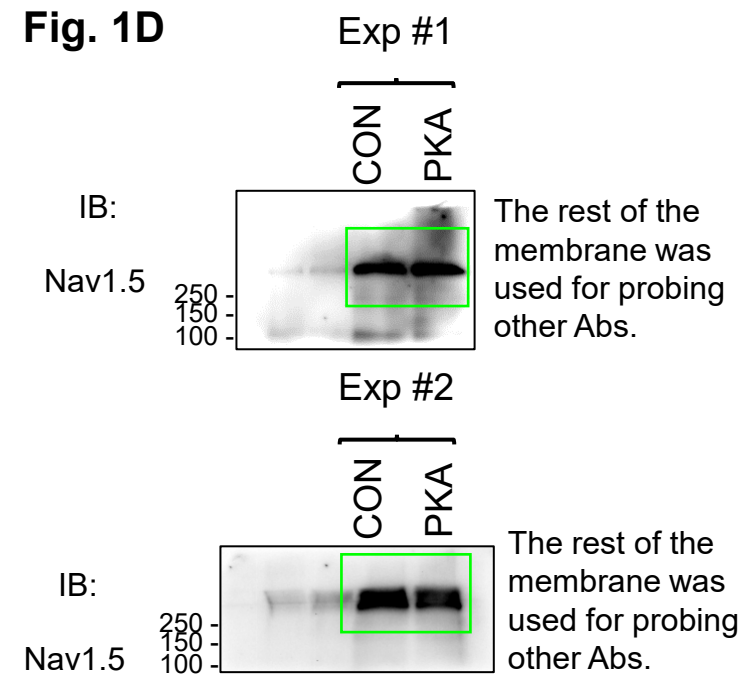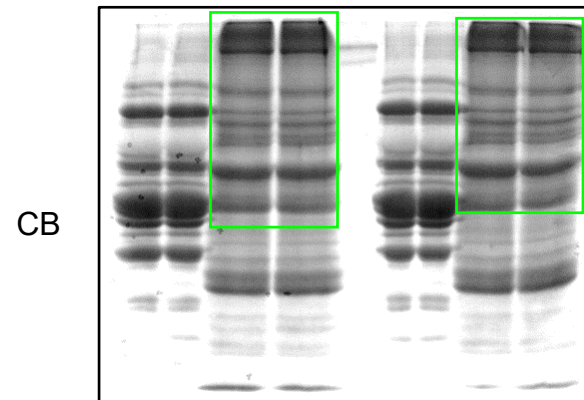

Supplement: SourceData F1 — is the source file for Fig. 1. [file JGP_202313436_SourceDataF1.pdf]

**Fig. 4A**

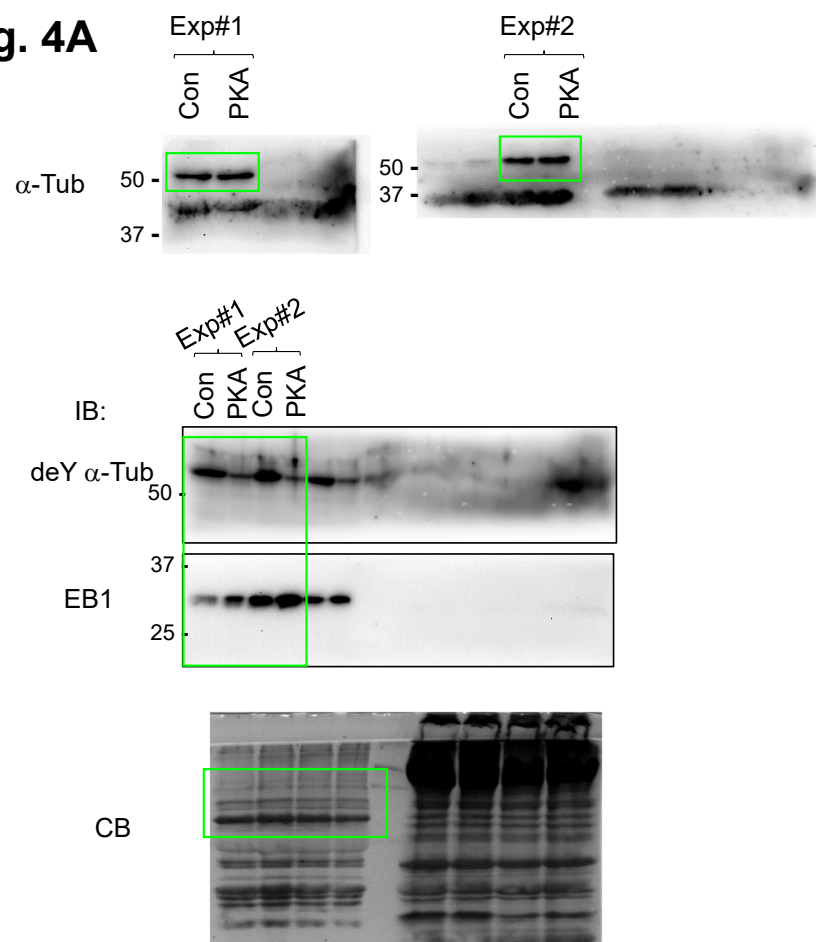

**Source Data for Fig. 4**

Supplement: SourceData F4 — is the source file for Fig. 4. [file JGP_202313436_SourceDataF4.pdf]

**Fig. 9A**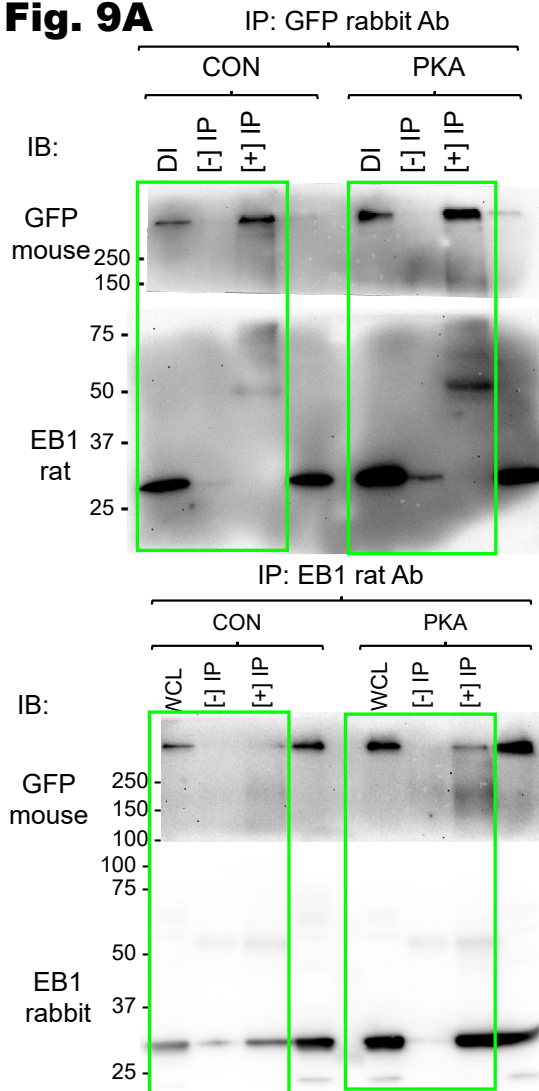**Fig. 9B**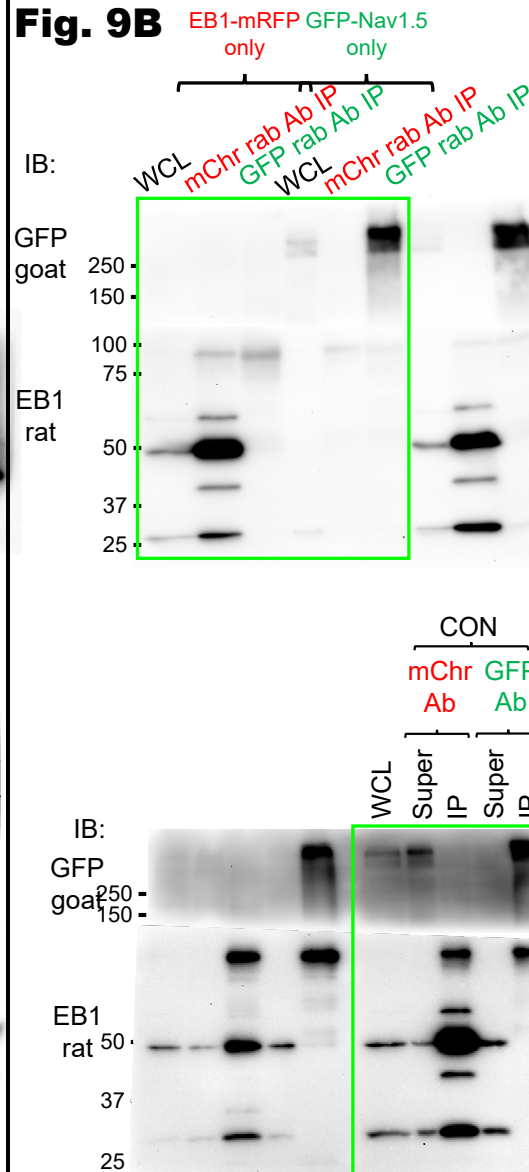**Fig. 9C**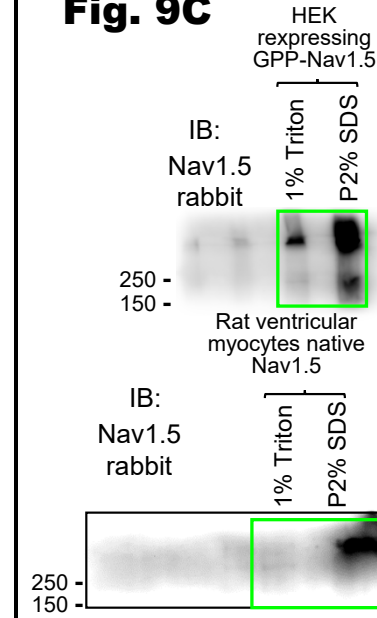**Fig. 9D**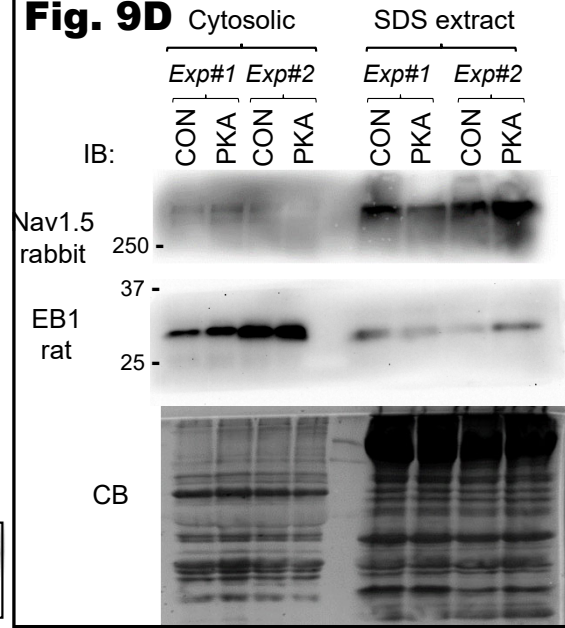**Source Data for Fig. 9**

Supplement: SourceData F9 — is the source file for Fig. 9. [file JGP_202313436_SourceDataF9.pdf]

**Fig. 11A**

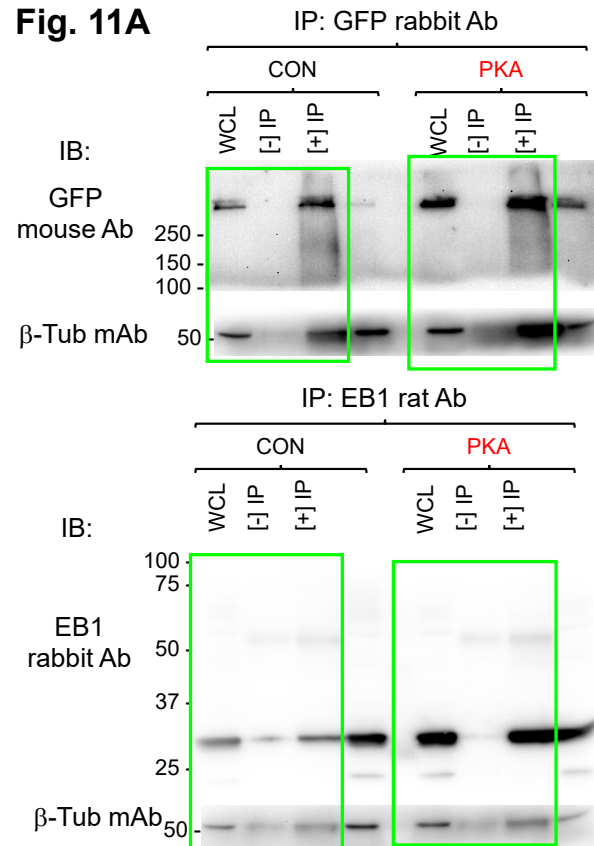

**SOURCE DATA for Fig. 11**

Supplement: SourceData F11 — is the source file for Fig. 11. [file JGP_202313436_SourceDataF11.pdf]

**Fig 12A**

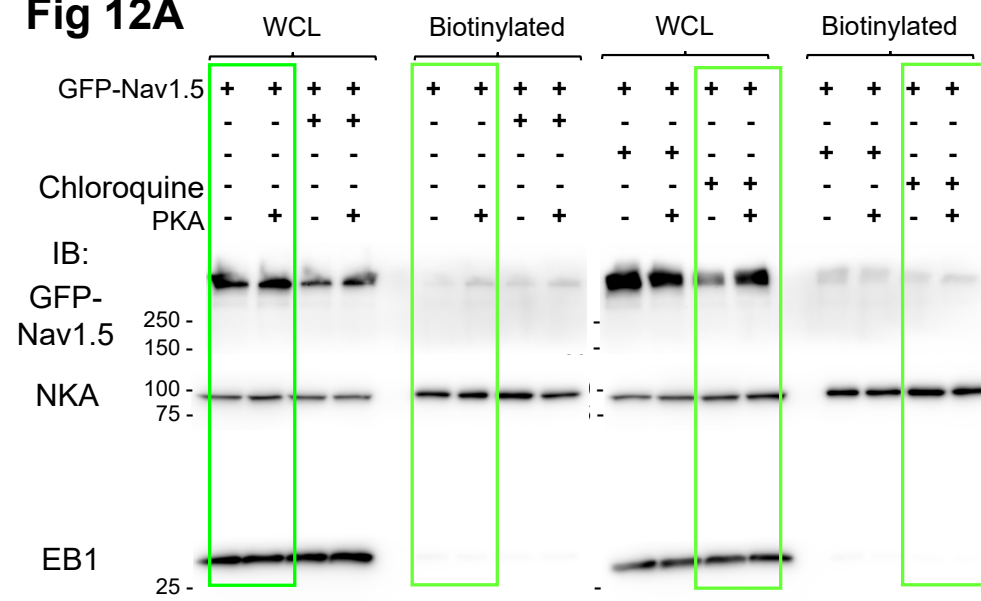

**SOURCE DATA FOR Fig. 12**

Supplement: SourceData F12 — is the source file for Fig. 12. [file JGP_202313436_SourceDataF12.pdf]

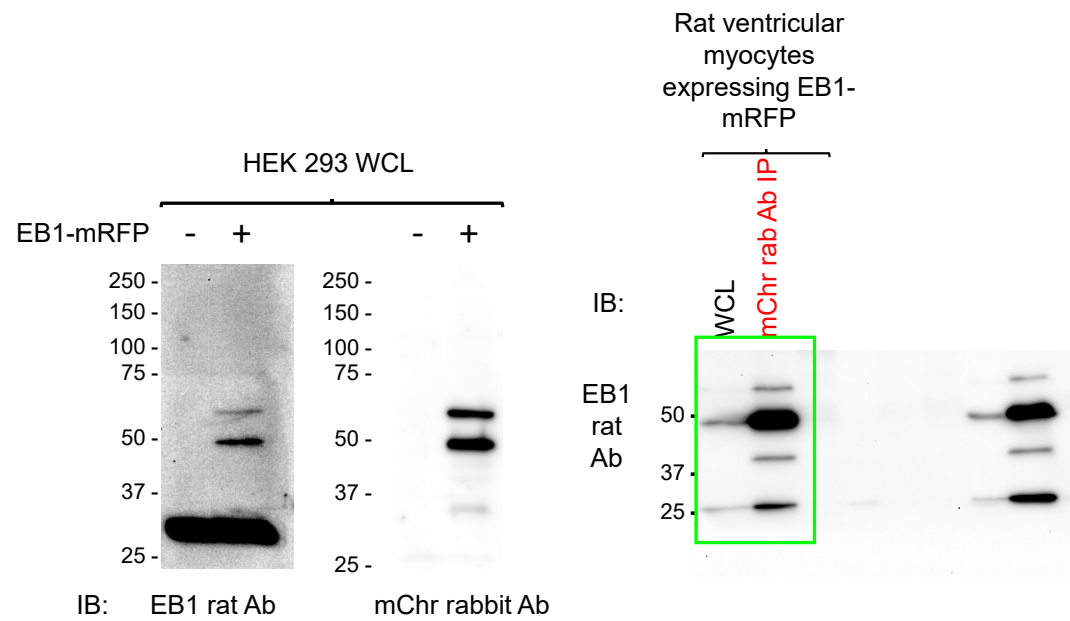

SOURCE DATA FOR Fig. S4

Supplement: SourceData FS4 — is the source file for Fig. S4. [file JGP_202313436_SourceDataFS4.pdf]
